# Repressible promoters – A novel tool to generate conditional mutants in *Pichia pastoris*

**DOI:** 10.1186/1475-2859-12-6

**Published:** 2013-01-24

**Authors:** Marizela Delic, Diethard Mattanovich, Brigitte Gasser

**Affiliations:** 1 Department of Biotechnology, University of Natural Resources and Life Sciences Vienna (BOKU), Muthgasse 18, 1190, Vienna, Austria; 2 Austrian Centre of Industrial Biotechnology (ACIB GmbH), 1190, Vienna, Austria

**Keywords:** Repressible promoter, *Pichia pastoris*, Promoter exchange

## Abstract

**Background:**

Repressible promoters are a useful tool for down-regulating the expression of genes, especially those that affect cell viability, in order to study cell physiology. They are also popular in biotechnological processes, like heterologous protein production.

**Results:**

Here we present five novel repressible *Pichia pastoris* promoters of different strength: P_*SER1*_, P_*MET3*_, P_*THR1*_, P_*PIS1*_ and P_*THI11*_. eGFP was expressed under the control of each of these promoters and its fluorescence could be successfully decreased in liquid culture by adding different supplements. We also expressed the essential genes with different native promoter strength, *ERO1* and *PDI1*, under the control of two of the novel promoters. In our experiments, a clear down-regulation of both repressible promoters on transcriptional level could be achieved. Compared to the transcript levels of these two genes when expressed under the control of their native promoters, only *ERO1* was significantly down-regulated.

**Conclusion:**

Our results show that all of the novel promoters can be used for repression of genes in liquid culture. We also came to the conclusion that the choice of the repressible promoter is of particular importance. For a successful repression experiment it is crucial that the native promoter of a gene and the repressible promoter in its non-repressed state are of similar strength.

## Background

The methylotrophic yeast *Pichia pastoris* is a favored microorganism for the production of heterologous proteins in biotechnology. It has gained its popularity, because this yeast is easy to manipulate, can grow to high cell densities on cheap cultivation media and is well suited for the production of secreted proteins [1,2]. Recently *P. pastoris* has also gained increasing interest as a model for cell biological studies of higher eukaryotic cells [3-5].

For biotechnological processes as well as for fundamental research, controlling of gene expression is required. In the industrial production of proteins inducible or repressible promoters are desirable, as they enable the separation of the cell growth phase from the protein production phase. Inducible/repressible promoters are also a useful tool for studying the function of essential genes, where the cells would not survive a complete knock-out.

Studies on repressible promoters are quite rare in yeast, and have mostly been performed with *Saccharomyces cerevisiae*. There, the most prominent candidates are the promoters of the genes *MET3* (repressed by methionine), *PHO5* (down-regulated by inorganic phosphate), *CUP1* (responsive to Cu^2+^ ions), and the *GAL* genes (active in presence of galactose, inactive with glucose) [6]. However, the commonly used regulable promoters in *P. pastoris* are those of the methanol utilization pathway, which enable high expression levels. These promoters are well suited for recombinant protein production, but not for studying gene function in cell biology. The best known is the alcohol oxidase 1 (*AOX1)* promoter which is activated on methanol and repressed on glucose [7]. As the switch from glucose to methanol as carbon source leads to massive physiological changes in the cell, this class of promoters is unfavorable for cell biological research. Therefore the demand is rising for alternatives. Recently, we discovered novel *P. pastoris* promoters by cultivating strains on different carbon sources (glycerol, glucose and methanol). Among them, especially P_*THI11*_ has been shown to be independent from regulation by the tested carbon sources, but was repressed in presence of thiamine [8]. Based on literature search in *S. cerevisiae*[9-12] and own microarray experiments in *P. pastoris* we identified other potential repressible promoters (P_*PIS1*_, P_*THR1*_, P_*SER1*_ and P_*MET3*_), which all could be of interest for studying physiology of the cell.

## Results and discussion

### Determination of promoter activities with eGFP as reporter protein

In this study we tested five different promoters for their ability to be repressed under certain cultivation conditions. Promoter sequences of the genes *THI11*, *SER1*, *MET3*, *THR1* and *PIS1* (Table 1) were amplified from the *P. pastoris* genomic DNA (wild type X-33) and fused to the gene of the enhanced green fluorescent protein (eGFP) as a reporter. *P. pastoris* strains expressing eGFP under the control of these promoters were cultivated in the synthetic M2 medium either without or with respective repressing supplements, in order to test the regulation of the chosen promoters by these compounds. Fluorescence of all strains was measured by flow cytometry during the exponential growth phase.

**Table 1:** Gene functions related to the promoters used in this work (from the Saccharomyces Genome Database)

**Gene**	**Function**	**Inducible/repressible**
*THI11*	Protein involved in synthesis of the thiamine precursor hydroxymethylpyrimidine (HMP)	Repressible with addition of thiamine
*THR1*	Conserved protein required for threonine biosynthesis (homoserine kinase)	Repressible with addition of L-threonine, L-valine, L-leucine and L-isoleucine
*MET3*	ATP sulfurylase, catalyzes the primary step of intracellular sulfate activation, essential for assimilatory reduction of sulfate to sulfide, involved in methionine metabolism	Repressible with addition of L-methionine
*SER1*	3-phosphoserine aminotransferase, catalyzes the formation of phosphoserine from 3-phosphohydroxypyruvate, required for serine and glycine biosynthesis	Repressible with addition of L-serine
*PIS1*	Phosphatidylinositol synthase, required for biosynthesis of phosphatidylinositol, which is a precursor for polyphosphoinositides, sphingolipids, and glycolipid anchors for some of the plasma membrane proteins	Repressible with addition of zink

The *P. pastoris* promoter P_*MET3*_ responded to addition of L-methionine and the initial fluorescence was fully down-regulated in the presence of this amino acid. The ability of the *MET3* promoter to be repressed by addition of methionine was already shown for *S. cerevisiae* and *Ashbia gossypii*[11,12]. Dünkler and Wendland have shown that this promoter was almost fully inactive during a period of 5 h, when the medium was supplemented with 2.5 mM methionine. Usually, the *MET3* promoter was used for short-term studies on cell physiology in *S. cerevisiae* as shown in Dünkler and Wendland [12]. We used P_*MET3*_ for the regulation of the promoter expression in liquid culture cultivations for a period of ~20 h with 10 mM methionine. The higher methionine concentration is possibly the explanation for the longer repression period of this promoter.

**Figure 1:**
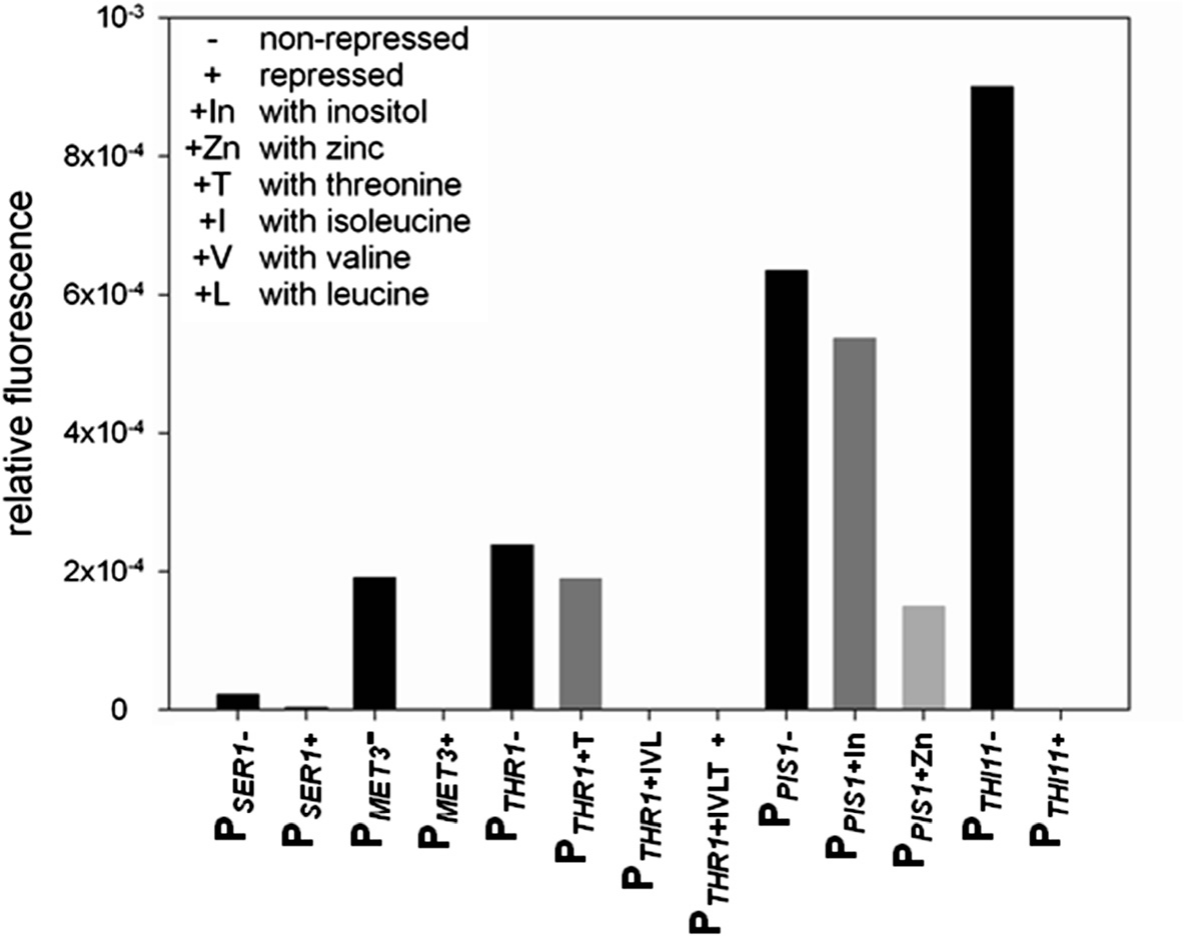
**Regulation of different repressible promoters in liquid culture.** Relative eGFP fluorescence per cell size under non-repressing (black) and repressing (gray) conditions using the promoters P_*SER1*_, P_*MET3*_, P_*THR1*_, P_*PIS1*_ and P_*THI11*_ with different supplements. The relative standard deviation of eGFP fluorescence between replicates was between 10 and 20%.

The *SER1* promoter can be repressed by addition of L-serine to the medium, but only to 30% under the cultivation conditions of these experiments. In case of the thiamine repressible promoter P_*THI11*_ there was no residual fluorescence in presence of thiamine after background correction with the fluorescence of the wild type X-33 (Figure 1).

Addition of L-threonine in order to reduce eGFP fluorescence when expressed under the control of the P_*THR1*_ promoter resulted only in a slight decrease of the signal. As the L-threonine biosynthesis pathway branches into the synthesis of L-isoleucine, L-valine and L-leucine, we added these three amino acids to the culture and combined them in the following cultivations with L-threonine. The highest repression of this promoter is achieved, when all four amino acids are present in the cultivation medium, resulting in a residual fluorescence of ~0% (Figure 1).

Contrary to published data for the *S. cerevisiae PIS1* promoter [13], the *P. pastoris* P_*PIS1*_ promoter responded only slightly to addition of inositol to the culture. As reported for *S. cerevisiae*[9,10], we could achieve a down-regulation of ~49% of the P_*PIS1*_ promoter by supplementing the medium with zinc sulfate (Figure 1).

The results above show that all tested *P. pastoris* promoters are repressible to a certain level under defined cultivation conditions. Under repressing conditions, residual fluorescence of the reporter protein eGFP could be detected only in case of P_*SER1*_ and P_*PIS1*_, which means that the other tested promoters were almost fully repressed under these conditions. In this study we tested only one concentration of all supplements. We do not rule out that an increased concentration of the amino acids, zinc sulfate, or other additional components would not cause different regulation of the chosen promoters. Also different cultivation conditions or cultivation time could influence the efficiency of down-regulation.

### Comparison of promoter strength

**Figure 2:**
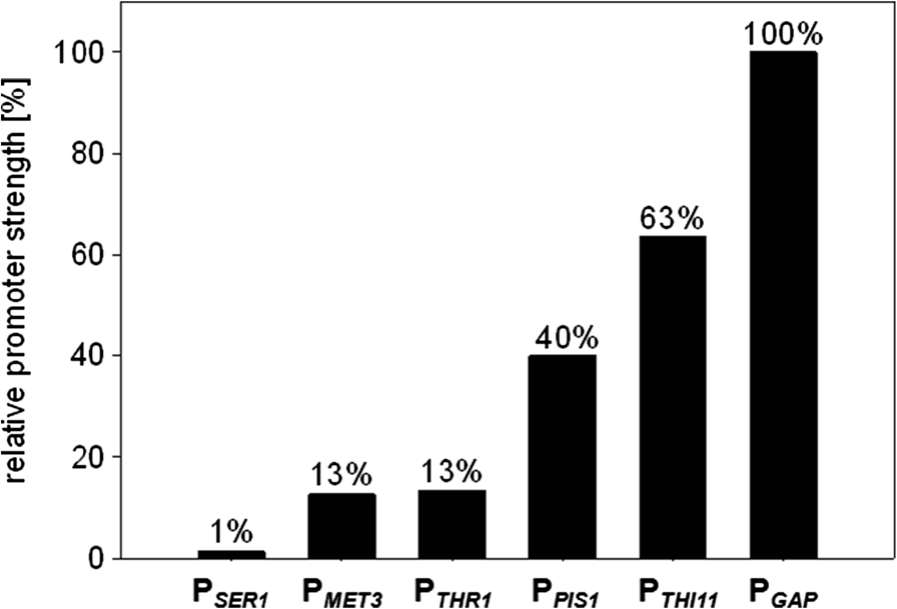
**Comparison of promoter strength of the repressible promoters under non-repressing conditions compared to P**_***GAP***_**.** eGFP fluorescence of the strong and constitutive GAP promoter (P_*GAP*_) is set as 100% and the other promoters, in their non-repressed form, are shown as relative values (in %) compared to P_*GAP*_.

In this study, we were not only interested in the transcriptional regulation of the five promoters that were chosen, but also in their relative strength in the non-repressed form in comparison to each other, and to the strong constitutive *P. pastoris* GAP (glyceraldehyde-3-phosphate dehydrogenase) promoter. The fluorescence of the promoter P_*GAP*_ was set to the value 100%, and that of the others was normalized to this value. The promoters of the genes *THR1* and *MET3* appear to have similar strength (13% of P_*GAP*_) and show ~10 times higher expression of eGFP than the P_*SER1*_ promoter under the described cultivation conditions. Under non-repressing conditions, the P_*PIS1*_ promoter was determined to be three times stronger than P_*THR1*_ and P_*MET3*_. The promoter of *THI11* was the strongest among the analyzed repressible promoters according to eGFP fluorescence measurements. The P_*THI1*_ promoter exhibits ~63% of the GAP promoter strength under the described cultivation conditions. The fluorescence achieved with the weakest (P_*SER1*_), compared to the strongest (P_*THI11*_) promoter, is almost 50 times lower (Figure 2).

### Example: Expression analysis of different essential genes under the control of novel repressible promoters

Exemplarily, we combined two of the tested promoters with genes that are involved in oxidative protein folding, *ERO1* and *PDI1*. Their deletions have been shown to affect the viability of the yeast *S. cerevisiae*[14]. These two genes exhibit promoters of different strength, *ERO1* having a weaker native promoter (~14% of the strong GAP promoter) than *PDI1* (~35% of the GAP promoter) in *P. pastoris* ([3] and own unpublished data).

**Figure 3:**
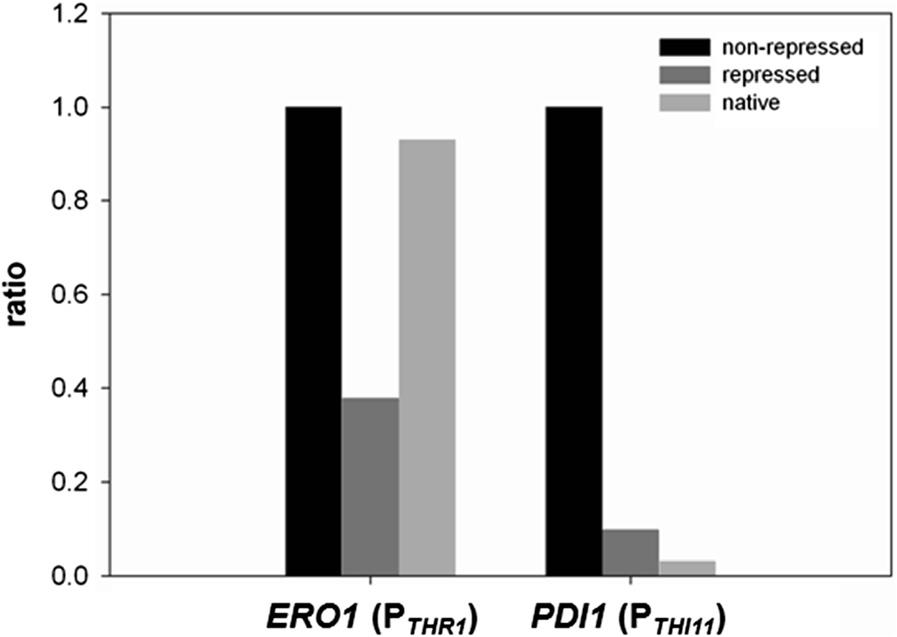
**Down-regulation of expression in liquid culture.** Relative transcript levels of *ERO1* and *PDI1* under control of the repressible promoters P_*THR1 *_and P_*THI11*_ in non- repressing and repressing conditions, as determined by qRT-PCR. They are compared to the relative transcript level of the respective native promoter.

We combined P_*THR1*_ with *ERO1* and P_*THI11*_ with *PDI1* by replacing the native gene promoter with the respective repressible promoter sequence, and determined relative transcript levels from liquid cultivations with and without supplements after 20 h of exponential growth (as described in Methods). In each case addition of the repressing supplements caused a reduction of the transcription of the respective gene. The measured residual expression was ~10% for *PDI1* under the control of P_*THI11*_ and ~40% for *ERO1* when expressed under the control of P_*THR1*_ (Figure 3). Compared to the native promoter, *ERO1* was down-regulated to approximately 40% by adding a mixture of threonine, leucine, isoleucine and valine, whereas *PDI1* did not undergo a transcriptional down-regulation upon promoter exchange. As P_*THI11*_ is more than 30 times stronger than the native promoter of *PDI1*, this repressible promoter turned out not to be suitable for this kind of experiment, as in the end *PDI1* was overexpressed and not down-regulated.

From these results we can conclude that not only the strength of the repressible promoter in its non-repressed state, but also in its repressed state is of great importance. This should be taken into account when a repressible promoter is chosen for down-regulation of the transcription of a gene of interest. In this case both, the repressible and the native promoter of this gene should be of comparable strength.

## Conclusion

Repressible promoters are an attractive tool for studying effects of conditionally down-regulated expression of genes that cannot be deleted. These promoters are mostly easy to handle and can be regulated with addition of few supplements. Here we present five repressible *P. pastoris* promoters of different strength. Each of the analyzed promoters can be successfully down-regulated or completely turned off in long term cultivations in liquid culture. The promoter strength ranged from a very weak promoter P_*SER1*_ to a strong promoter P_*THI11*_ with comparable expression levels to the strong *P. pastoris* P_*GAP*_ promoter.

In our experiments, we succeeded to down-regulate the transcriptional expression of two essential genes, *ERO1* and *PDI1*, when they were expressed under the control of two different repressible promoters P_*THR1*_ and P_*THI11*_. Remarkably, only *ERO1* was really down-regulated below its native transcriptional level, when the results of expression levels were compared to those of the wild type strain. These experiments demonstrated clearly how important the choice of the repressible promoter is, as the residual expression levels of these two genes after down-regulation differed immensely. The repressible promoter should be comparable in its non-repressed state to the native promoter of a gene of interest.

We could also observe a significant transcript level down-regulation of the promoters P_*MET3*_ and P_*SER1*_ when we cultivated the strains in complex medium (modified YPD medium), but not of the promoter P_*THR1*_ (data not shown).

## Methods

### Promoter study

*P. pastoris* (wild type strain X-33) promoters (1000 bp upstream of the ATG start codon) of the genes *THI11* (PAS_chr4_0065, promoter P_*THI11*_)*, MET3* (PAS_chr1-4_0253, promoter P_*MET3*_)*, SER1* (PAS_chr3_0566, promoter P_*SER1*_), *THR1* (PAS_chr3_0899, promoter P_*THR1*_) and *PIS1* (PAS_chr2-1_0694, promoter P_*PIS1*_) were amplified from X-33 genomic DNA and cloned into the pPuzzle vector [8] with eGFP (enhanced green fluorescent protein, AEI54555) as reporter gene (see Table 2). The Zeocin resistance marker was flanked by loxP sites. The vectors were integrated into the *AOX1* terminator locus of the *P. pastoris* genome after linearization with *Asc*I in the respective sequence. After transformation by electroporation positive transformants were selected on YPD agar plates with 25 mg/L Zeocin.

**Table 2:** Primers used for cloning of the expression vectors

**Primer name**	**Primer sequence**
P_*MET*__fw (*BstX*I):	TAGA**CCAAGGCCTTGG**AATTCAGGCAACAGGACC
P_*MET*__rv (*Sbf*I):	AATA**CCTGCAGG**TTCTTTCTGAGTTGGTTTCC
P_*THR*__fw (*BstX*I):	TAGA**CCAAGGCCTTGG**TTCTTCAGAATGGGAACTAG
P_*THR*__rv (*Sbf*I):	AATA**CCTGCAGG**TGTCTGCCACACAAATTAA
P_*SER*__fw (*BstX*I):	TAGA**CCAAGGCCTTGG**CAGCAAATAATTAGCAGCC
P_*SER*__rv (*Sbf*I):	AATA**CCTGCAGG**TGTATTATATGGTTAGTTCAAGATG
P_*PIS*__fw (*BstX*I):	TAGA**CCAAGGCCTTGG**GTAACGAGGCTAAAAGTTTTTGC
P_*PIS*__rv (*Sbf*I):	AATA**CCTGCAGG**TGCAGGTGGACTATCTAGAGACAAG
P_*THI*__fw (*BstX*I):	TAGA**CCAAGGCCTTGG**CATCTTTTCAGCTTCATCGTCAG
P_*THI*__rv (*Sbf*I):	AATA**CCTGCAGG**ATGATTTATTGAAGTTTCCAAAGTTG
P_*ERO*__fw (*Asc*I):	ATTA**GGCGCGCC**AAGGGAACCCATTTTCTTCG
P_*ERO*__rv (*Apa*I):	ATTA**GGGCCC**GGTAGTGGAACAGCAAGATGG
P_*PDI*__fw (*Asc*I):	ATTA**GGCGCGCC**ATTCCGGAGATTCACATTGC
P_*PDI*__rv (*Apa*I):	ATTA**GGGCCC**TCATCGGGCAGTTCTTTCTT

### Promoter replacement

**Figure 4:**
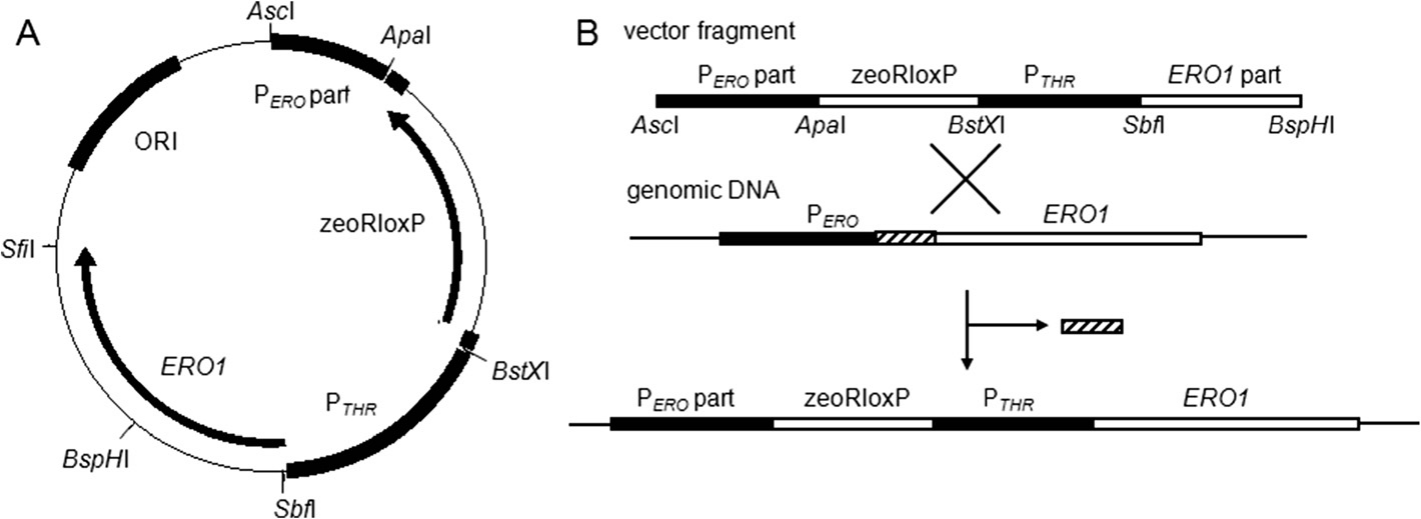
**Promoter replacement strategy.** (**A**) The pPuzzle vector was used as backbone for cloning the promoter exchange cassette of the two genes *ERO1* and *PDI1*. P_*ERO*_ part is a fragment of ~500 bp of the native *ERO1* promoter, about −700 to −200 bp upstream of the ATG. Zeocin was used as selection marker, where loxP sites could be used for marker recycling. (**B**) After digestion with *Asc*I and *BspH*I the promoter exchange cassette was transformed into *P. pastoris*. It interacted there with the respective genomic DNA by homologous recombination and replaced the native promoter of the respective gene. The promoter exchange cassette integrated into the genomic DNA, whereby 200 bp of the native promoter were excised.

*P. pastoris* genes *ERO1* (PAS_chr1-1_0011) and *PDI1* (PP7435_Chr4-0183), and 500 bp of their respective promoter regions (starting −200 bp upstream from the ATG) were amplified by PCR from X-33 genomic DNA (primers are listed in Table 2). The expression cassette was generated by flanking the resistance cassette Zeocin 5’ with ~ 500 bp of native promoter sequence of the gene of interest, and 3’ by the repressible promoter, which was followed by the *P. pastoris* gene of interest (Figure 4A). We combined the repressible promoter P_*THR1*_ with *ERO1* and P_*THI11*_ with *PDI1*. After digestion with *Asc*I and *BspH*I the transformation of the *P. pastoris* wild type strain X-33 was performed by electroporation. The repressible promoter cassette should interact with the genomic DNA in the cell and replace the respective promoter of the gene of interest (Figure 4B) [15].

### Cultivation medium

M2 minimal medium contained per liter: 20 g of glucose, 20 g of citric acid, 3.15 g of (NH_4_)_2_HPO_4_, 0.03 g of CaCl_2_.2H_2_O, 0.8 g of KCl, 0.5 g of MgSO_4_.7H_2_O, 2 mL of biotin (0.2 g L^-1^), 1.5 mL of trace salts stock solution. The pH was set to 5.0 with 5 M KOH solution. Trace salts stock solution contained per liter: 6.0 g of CuSO_4_.5H_2_O, 0.08 g of NaI, 3.0 g of MnSO_4_.H_2_O, 0.2 g of Na_2_MoO_4_.2H_2_O, 0.02 g of H_3_BO_3_, 0.5 g of CoCl_2_, 20.0 g of ZnCl_2_, 5.0 g of FeSO_4_.7H_2_O, and 5.0 mL of H_2_SO_4_ (95-98% w/w).

### Cultivation conditions

All analyzed clones were grown over night in 5 mL M2 medium as pre-culture. Main cultures were inoculated to an OD_600_ = 0.1 and incubated in 100 mL shake flasks at 28°C with 170 rpm (rotations per minute). Fluorescence of the cells was measured after 24 h of cultivation. All clones were supplemented two more times (after ~12 h) with the respective components (concentration as described below).

For comparing the non-repressed and repressed state of a promoter, cultivations of same clones in the M2 medium with and without supplements were performed. For repressing the promoters following concentrations of supplements were added to the medium: for P_*THI11*_ 10 mM of thiamine hydrochloride (Merck), for P_*SER1*_ 10 mM of L-serine (Serva), for P_*MET3*_ 10 mM of L-methionine (Serva), for P_*THR1*_ 10 mM of L-threonine (Serva), L-leucine (Serva), L-isoleucine (Serva) and L-valine (Serva), and for P_*PIS1*_ 100 μM inositol or 1.5 μM zinc sulfate (Merck).

For selection on plates, 2% agar was added to the M2 medium and the pH was set to ~ 7.

### Fluorescence measurement with flow cytometry

1 mL cells of an OD_600_ of 0.4 were harvested by centrifugation and resuspended in 2 mL PBS. Flow cytometry analysis was performed using a FACS Canto (Becton Dickinson) with these settings: excitation wavelength at 488 nm, emission wavelength at 530 nm (green filter, 525–550 nm). Measured fluorescence was referred to the cell size.

### Isolation of total RNA from *P. pastoris*, reverse transcription of mRNA and determination of transcript levels by quantitative real time PCR

RNA isolation, cDNA synthesis and measurement of mRNA transcript levels using real time PCR was performed as described in [8]. Samples were taken after 20 h of cultivation. Actin (*ACT1*) was used as reference gene for normalization. Each gene was correlated to actin as internal control, and the respective non-repressed strain served as the reference strain for each relative transcript level determination with the delta-delta C_t_ method [16]. RT PCR primers are listed in Table 3.

**Table 3:** **Real time PCR primers for *ERO1 *and *PDI1***

**Primer name**	**Primer sequence**	**Amplicon size (bp)**
ERO_fw:	GTTGGAAAAGCCGCATATAAACAAAACA	141
ERO_rv:	CAGCTTGGGCAAAGTCCTGTAAGAGTTC	
PDI_fw:	GGAAAGGCCCACGATGAAGTTGTC	140
PDI_rv:	GCATCCTCATCATTGGCGTAAAGAGTAG	
ACT_fw:	CCTGAGGCTTTGTTCCACCCATCT	148
ACT_rv:	GGAACATAGTAGTACCACCGGACATAACGA	

## Competing interests

The authors declare to have no competing interests.

## Authors’ contributions

MD contributed to selecting the promoter sequences, performed the experimental work and drafted the manuscript. DM coordinated the project and contributed to drafting the manuscript. BG designed the study and contributed to drafting the manuscript. All authors read and approved the final manuscript.
